# Speech monitoring and phonologically-mediated eye gaze in language perception and production: a comparison using printed word eye-tracking

**DOI:** 10.3389/fnhum.2013.00818

**Published:** 2013-12-10

**Authors:** Hanna S. Gauvin, Robert J. Hartsuiker, Falk Huettig

**Affiliations:** ^1^Department of Experimental Psychology, Ghent UniversityGhent, Belgium; ^2^Psychology of Language Department, Max Planck Institute for PsycholinguisticsNijmegen, Netherlands; ^3^Donders Institute for Brain, Cognition and Behavior, Radboud UniversityNijmegen, Netherlands

**Keywords:** language production, speech perception, perceptual loop theory, verbal self-monitoring, speech prediction

## Abstract

The Perceptual Loop Theory of speech monitoring assumes that speakers routinely inspect their inner speech. In contrast, Huettig and Hartsuiker (2010) observed that listening to one's own speech during language production drives eye-movements to phonologically related printed words with a similar time-course as listening to someone else's speech does in speech perception experiments. This suggests that speakers use their speech perception system to listen to their own overt speech, but not to their inner speech. However, a direct comparison between production and perception with the same stimuli and participants is lacking so far. The current printed word eye-tracking experiment therefore used a within-subjects design, combining production and perception. Displays showed four words, of which one, the target, either had to be named or was presented auditorily. Accompanying words were phonologically related, semantically related, or unrelated to the target. There were small increases in looks to phonological competitors with a similar time-course in both production and perception. Phonological effects in perception however lasted longer and had a much larger magnitude. We conjecture that this difference is related to a difference in predictability of one's own and someone else's speech, which in turn has consequences for lexical competition in other-perception and possibly suppression of activation in self-perception.

## Introduction

It has been estimated that during speech production, in both conversation and monolog, one out of ten utterances are subject to revision (Nakatani and Hirschberg, 1994). These revisions partly take place after articulation, but there is reason to believe that speech error monitoring also takes place before articulation. Evidence for such a pre-articulatory speech production monitor comes from our capacity to produce extremely fast corrections, even before the error is fully produced (Levelt, 1989). Additionally, corrections are still made when auditory feedback is disrupted, for instance by masking overt speech (Postma and Noordanus, 1996). And even when speech is only produced internally, production errors are still reported (Oppenheim and Dell, 2008). Such pre-articulatory monitoring might affect patterns of speech errors, as shown in studies where participants produce fewer word slips when this slip would result in a taboo utterance or a nonsense word (Baars et al., 1975; Motley et al., 1982; Hartsuiker et al., 2005; Nooteboom and Quené, 2008; Dhooge and Hartsuiker, 2012). In sum, there appears to be an external monitoring channel that monitors speech after articulation, and an internal monitoring channel that monitors speech before articulation.

There are several theories on how the internal speech monitoring mechanism works. One influential theory, the Perceptual Loop Theory (Levelt, 1989) holds that during speech production copies of the created speech plan are sent via internal loops to the speech comprehension system. This takes place at two levels of production, namely the preverbal message (conceptual loop) and the articulatory buffer (inner loop). Wheeldon and Levelt (1995) further suggested that a phonemic representation is fed back to the comprehension system. In essence, the perceptual loop theory assumes one speech monitoring mechanism for both internally and externally produced speech that is based on speech perception. On the other hand, production-based approaches assume monitoring systems that are extrinsic to the perception system (see Postma, 2000, for a review). For instance, several authors have recently suggested monitoring systems based on *forward models* (e.g., Hickok, 2012; Pickering and Garrod, 2013); the speaker creates a prediction (or forward model) of the expected utterance and compares it to the actual produced speech. Additionally, Nozari et al. (2011) argue that a monitor that assesses the amount of conflict within representational layers (i.e., whether only a single representational unit is highly active or whether several units are highly active) would be diagnostic of error trials. All such production monitoring accounts have in common that internal monitoring would be based on mechanisms that are internal to the production system, rather than on the perception of inner speech.

Empirical evidence on the systems responsible for inner monitoring is scarce and inconsistent. Studies with brain-damaged patients have shown dissociations between comprehension abilities and self-monitoring abilities, a finding that appears inconsistent with the perceptual loop theory. A particular striking study (Marshall et al., 1985) reports the case of a patient with the inability to ascribe meaning to spoken words (and even everyday sounds) indicating a profound disorder of comprehension, who nevertheless initiated self-corrections in her speech. On the other hand, a reaction time study with healthy young adults (Özdemir et al., 2007) reported that response times to phoneme monitoring (e.g., push the button if the name of a target picture contains a particular phoneme) depended on the so-called perceptual uniqueness point, a variable that affects speech perception but not production. However, neither type of evidence is fully convincing: it is possible that patients with good monitoring despite poor comprehension have a comprehension deficit at a relatively early perceptual stage and so accurately perceive inner speech (Hartsuiker and Kolk, 2001). Moreover, it is possible that the phoneme-monitoring task is a very poor model of monitoring in overt production where perception of inner speech might interact with the perception of overt speech (Vigliocco and Hartsuiker, 2002).

In a more recent test of the role of the speech perception system in the internal channel of speech monitoring, Huettig and Hartsuiker (2010) conducted an object naming study using a printed word version of the visual world paradigm. In this version of the visual world paradigm, participants view a display of printed words (typically four words, one in each corner) and listen to spoken language. Looks to each of the words are recorded as a function of the spoken stimuli. For instance, McQueen and Viebahn (2007) showed that participants are more likely to look at printed words with names matching the onset of the concurrent spoken word (e.g., the Dutch word *buffer* when hearing *buffel* “buffalo”) than to printed words that are phonologically different or which match at word offset (e.g., *motje* “moth” for *rotje* “firecracker”). These results are consistent with experiments using the picture version of the visual world paradigm (e.g., Allopenna et al., 1998) and several other methods (phoneme monitoring, Connine et al., 1997; cross-modal priming, Marslen-Wilson and Zwitserlood, 1989). In Huettig and Hartsuiker's production study, participants named visual objects that were presented together with three printed words. These printed words were phonologically related, semantically related, or unrelated to the target. Consistent with earlier perception studies using printed words, there were no increased looks to semantic competitors when phonological competitors were co-present in the display (cf. Huettig and McQueen, 2007, 2011). However, similar to perception studies, phonological competitors received significantly more looks than phonologically unrelated distractors.

Importantly, the perceptual loop theory hypothesizes that the internal channel bypasses articulation and low-level auditory analysis. This allows for speech monitoring even before external speech. By skipping articulation, the target reaches the perceptual system between 250 ms before speech onset (Levelt, 1989; Hartsuiker and Kolk, 2001) and 145 ms before speech onset (Indefrey and Levelt, 2004). In other words, the perceptual loop theory predicts eye-fixations on printed phonological competitor words before participants produce their own speech. If we assume programming and eye-movements to take about 200 ms (Saslow, 1967), one expects the following results: eye-movements to the phonological competitor driven by internal speech should start between 50 ms before speech onset and 55 ms after; looks to the phonological competitor driven by external speech should start from 200 ms after speech onset. Huettig and Hartsuiker's (2010) results showed a phonological competitor effect in the same time range (300 ms post-articulation) as had been found in earlier perception studies (Huettig and McQueen, 2007), leading to the conclusion that listening to your own overt speech is the same as listening to someone else's speech. Because there was no indication that participants listen to their internal speech in overt speech production, their results argue against the perceptual loop theory for speech monitoring.

### Current study

There are both theoretical and practical reasons to revisit the claim that listening to self-produced speech is similar to listening to someone else's speech. First, while the similarity of the findings in Huettig and Hartsuiker (2010) and Huettig and McQueen (2007) is striking, they do not constitute a direct comparison between the modalities. Huettig and Hartsuiker only had a production condition, which was compared to results from a perception condition of an experiment with a different setup (Huettig and McQueen, 2007). For example, the former had a display with one target picture and three written competitors and the latter had a visual display with four printed words. Also target words were embedded in a sentence context in the perception condition, while the production experiment required only production of the target word. Thus, we believe a more direct comparison between production and comprehension is needed to establish whether listening to one's own production is really based on one's overt speech only^1^.

Second, and perhaps more interestingly, even if we take for granted that listening to one's own speech production is based on overt speech, there might still be differences between listening to one's own overt speech and to the overt speech of somebody else. This is because of an important difference between speech production and speech perception, namely that in speech production one can make much more accurate predictions of what speech will be produced than in perception. Pickering and Garrod (2013), for instance, hypothesized a role for prediction in both comprehension and production. By predicting upcoming words in speech perception, perception processes can take place much faster than if it were dependent on bottom-up processes only. However, predicting someone else's speech is of course associated with more uncertainty about upcoming speech than predicting one's own utterance, which is likely to affect patterns of overt visual attention in a visual world paradigm. In sum, given the sensitivity of eye-movements to linguistic predictions in visual world studies (e.g., Altmann and Kamide, 1999; Weber et al., 2006; Kamide, 2008; Kukona et al., 2011; Mani and Huettig, 2012), one might expect differences between eye-movements driven by hearing one's own voice vs. someone else's voice.

Thus, the current experiment investigated whether there is a role for the internal monitoring channel in speech production. By directly comparing phonologically-driven eye-movements in a visual world paradigm using matched speech perception and production conditions, we tested whether listening to one's own overt speech has similar perceptual effects to listening to someone else's overt speech. In both conditions participants were presented with a display with four written words and auditory stimuli consisting of only a noun in both the perception and production conditions.

## Materials and methods

### Participants

Forty participants (8 males, 32 females, aged 17–35) took part in exchange for course credits. Participants were recruited at the psychology department of Ghent University and were all native speakers of Dutch. All reported to have no dyslexia, no hearing problems, and correct or corrected to normal vision.

### Materials

**Figure 1:**
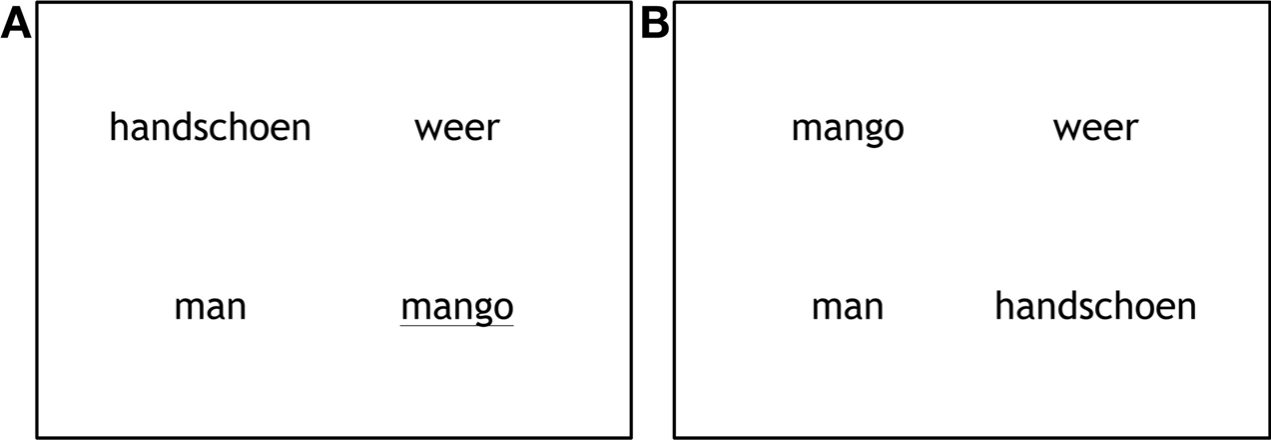
**Examples of the display in the production condition (A) and the perception condition (B)**.

We created 72 sets of visual displays. Each display showed four printed words (Font MS Trebuchet, size 20), each in a different quadrant of the screen (Figure 1). Each display consisted of one target word, one competitor word and two unrelated filler words. The words were randomly assigned to a quadrant per trial.

There were three conditions: in the semantic condition, the competitor was semantically related to the target; in particular, it came from the same category. In the phonological condition, the competitor shared the onset (from 1 up to 3 phonemes) with the target. In the neutral condition, the competitor was unrelated to the target. Each item was presented as target or competitor in one display, and presented as unrelated filler in another display; for example, targets and competitors in the phonological condition occurred as unrelated items in the neutral condition. Differences in looks to targets and competitors compared to unrelated items can therefore not be the result of intrinsic properties of the items. There were 24 trials in each condition (semantic, phonological, and neutral). The order of the trials was determined randomly. All stimuli were presented once in the production condition and once in the perception condition.

Most words in the phonological and semantic conditions were taken from Huettig and Hartsuiker (2010). For the phonological condition eight word pairs were created that shared a higher CV overlap between target and competitor compared to the original word pairs. In the semantic condition 11 new word pairs were created that (subjectively) had a stronger semantic relatedness. An overview of the stimuli can be found in Appendix A.

Participants filled out a questionnaire on their reading and auditory skills and signed a written consent form. The participants received written task instructions. Next the eye-tracking device was adjusted for each participant and calibrated. The experiment consisted of 12 blocks. At the beginning of each block a calibration of the eye-tracker was performed. During each block six trials of the production condition and six trials of the perception condition were presented consecutively. A picture of an ear (perception) or a mouth (production), displayed for 2000 ms, signaled the task. Each trial started with a fixation cross, followed by a 3000 ms display of the four written words. Displays were randomly assigned to each trial.

In the production trials the target word was underlined and was read out loud by the participant. In the perception condition participants heard the target word after a 200 ms preview of the display. After the experiment participants filled out similarity ratings for the semantic (how well do the words match?) and phonological word pairs (how similar are the word onsets?) on a 5-point scale, with 1 being “not at all” and 5 being “very much.” On average both semantic and phonological word pairs were rated as being between neutral and fairly similar. Semantic word pairs were rated as more similar (*M* = 3.52, *SE* = 0.077) than phonological word pairs (*M* = 2.96, *SE* = 0.083).

### Apparatus

Experiments were created in Experiment Builder 1.10.1 (SR Research Ltd. 2004–2010). Eye movements were recorded using an EyeLink 1000 eye-tracker. Speech in the production trials was recorded and speech onsets were measured manually in Praat (Boersma and Weenink, 2012).

## Results

Responses in which there was a hesitation (e.g., ‘eh’) or in which the response was produced after the display had disappeared (i.e., after 3000 ms) were excluded from analysis. No other outliers were excluded. This led to a total loss of 1.4% of all the production trials. Errors were fairly equally distributed among the three conditions. Naming latencies were around 1100 ms for all three conditions (Table 1).

**Table 1:** **Number of errors and mean speech onset**.

**Condition**	**Errors (%)**	**Onset (ms)**	***SD* onset**
Neutral	1.1	1160	315
Phonological	1.5	1118	296
Semantic	1.7	1169	302

### Analysis of fixation data

Fixation proportions to targets and related competitors were compared to an average of unrelated neutral competitor words in the respective conditions. The fixation proportions were calculated for 200 ms timeframes, until 1000 ms after speech onset for both production and perception conditions. To test whether visual attention in the production condition indeed precedes production analysis starts 200 ms before speech onset. Fixation proportions were normalized before averaging per condition using a log 10 transformation, and (as hypotheses were directional) compared in one-tailed paired *t*-tests, with alpha set at 0.05. Effect sizes reported are Cohen's *d.*

For ease of interpretation, reported means, and standard deviations in text, figures, and appendix, are the untransformed values.

### Perception

#### Fixations on target items

In the perception trials, eye-movements started to diverge toward the target words from around 200 ms after speech onset. Looks to the target in the neutral condition (*M* = 0.287 *SD* = 0.398) differentiated significantly from looks to the unrelated words (*M* = 0.232, *SD* = 0.360) between 200 and 400 ms after speech onset *t*_1(39)_ = 3.420, *p* < 0.001; *t*_2(23)_ = 4.34, *p* < 0.001; *d* = 0.145. Looks to the target in the phonological condition (*M* = 0.365, *SD* = 0.429) diverged significantly from the unrelated items (*M* = 0.182, *SD* = 0.333) between 400 and 600 ms after speech onset *t*_1(39)_ = 8.753, *p* < 0.001; *t*_2(23)_ = 6.794, *p* < 0.001; *d* = 0.476. Looks to the target in the semantic condition (*M* = 0.409, *SD* = 0.432) also diverged significantly from the unrelated items (*M* = 0.190, *SD* = 0.335) between 400 and 600 ms after speech onset *t*_1(39)_ = 10.308, *p* < 0.001; *t*_2(23)_ = 8.923, *p* < 0.001; *d* = 0.566.

#### Fixations on competitor items

Analysis of fixations to the neutral competitors revealed no significant differences at any time interval. For the semantic condition there was also no significant difference between looks toward the competitor and unrelated items in any of the timeframes.

**Figure 2:**
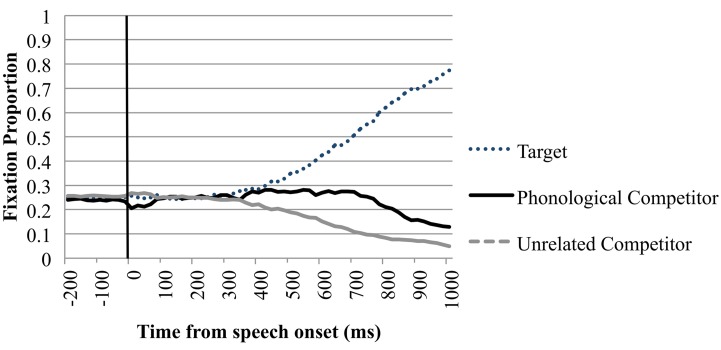
**Eye-movements in the phonological trials of the perception condition**. Proportion of fixations are sorted per quadrant and plotted as a function of time. Time point −200 is the onset of the display. Speech onset of the target word is at 0 ms, as indicated by the black line.

In the phonological condition there was a similar proportion of looks toward the phonological competitor and the unrelated items −200 ms until speech onset and in the timeframe from speech onset until 200 ms after this onset. Between 200 and 400 ms after speech onset we observed a 2.3% difference between looks toward the phonological competitor and the unrelated items. This difference did not reach statistical significance; the following timeframes however showed a more robust increase of this difference. This suggests that the phonological competitor effect started to arise in the 200–400 ms timeframe. Looks toward the phonological competitor (*M* = 0.270, *SE* = 0.385) diverged significantly from looks toward the unrelated items (*M* = 0.182, *SD* = 0.333) between 400 and 600 ms after speech onset *t*_1(39)_ = 5.743, *p* < 0.001; *t*_2(23)_ = 4.591, *p* < 0.001; *d* = 0.244. This effect increased and remained significant throughout the 1000 ms after speech onset. Figure 2 shows the time course probability plots for the phonological condition in the perception trials.

### Production

#### Fixations toward target items

At the start of the analysis, 200 ms before speech onset, fixations in the production condition were directed significantly more toward the target than to unrelated items in all three conditions. The difference between looks to the target items and unrelated items remained significant (*p* < 0.001) throughout the later timeframes.

#### Fixations on competitor items

In both the neutral and semantic condition there was never a significant difference of fixations between the competitors and unrelated items in all time bins.

**Figure 3:**
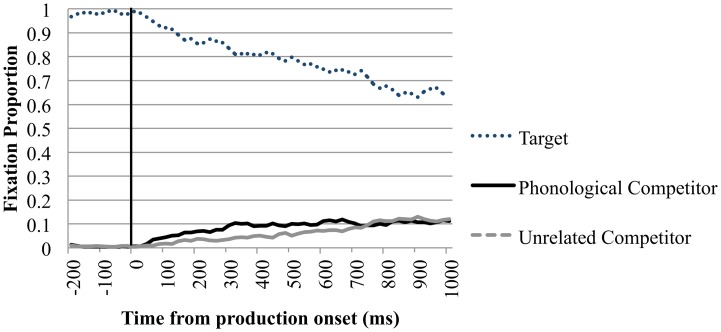
**Eye-movements in the phonological trials of the production condition**. Proportion of fixations are sorted per quadrant and plotted as a function of time. Time point 0 is the speech onset, as indicated by the black line.

Of main interest are the fixations on the phonological competitor. Importantly, between 200 ms before and 200 ms after speech onset, looks toward the phonological competitor did not differ significantly from looks to the unrelated items. Between 200 and 400 ms after speech onset looks to the phonological competitor (*M* = 0.076, *SD* = 0.247) diverged from the unrelated items (*M* = 0.054, *SD* = 0.214), *t*_1(39)_ = 1.717, *p* = 0.047; *t*_2(23)_ = 2.445, *p* = 0.012; *d* = 0.095. This timeframe is comparable to the 350–500 ms after speech onset in which eye-movements to phonological competitors were observed in Huettig and Hartsuiker (2010). In the 400–600 ms after speech onset there were more looks to the competitor in the phonological condition (*M* = 0.096, *SD* = 0.277) than to the unrelated items (*M* = 0.080, *SD* = 0.254) significant in the by-participant analysis *t*_1(39)_ = 2.110, *p* = 0.021; *t*_2(23)_ = 1.300, *p* = 0.104; *d* = 0.060. In the timeframes after 600 ms the phonological competitor did not attract more fixations than the unrelated items. Figure 3 shows the time course of fixation proportions for the phonological condition in the production trials.

In sum, in both production and perception trials eye-movements were directed more to phonological competitors than to unrelated printed words shortly after the critical onset of word perception or word production. The magnitude of the phonological competition effect however differed considerably between production and perception trials. An overview of the fixation proportions in the phonological condition can be found in Appendix B.

## Discussion

In the present experiment using the printed word version of the visual world paradigm, we observed more looks toward a phonological competitor in both speech perception and speech production. In line with previous studies (Huettig and McQueen, 2007; Huettig and Hartsuiker, 2010), there were no semantic effects using this version of the paradigm. The experiment allows for two main conclusions. First, phonological competition effects in production did not occur in the timeframe predicted by perceptual loop theory. Second, the magnitude and longevity of the effect was considerably larger in perception than in production. This suggests that overt speech is processed differently if it is produced by someone else rather than by oneself.

The speech production condition did not show a robust increase of eye-movements toward the phonological competitor shortly before or around speech onset. Thus, consistent with the results of Huettig and Hartsuiker's (2010), these findings do not support a role of speech perception for the monitoring of inner speech. Given the small effects in this study, however, we must acknowledge the possibility that the absence of early phonological competition effects reflects a lack of sensitivity to internal monitoring processes of our method. Future work should ideally use additional methods to provide converging evidence.

The difference in magnitude of looks toward the phonological competitor between speech production and speech comprehension is, in our view, the result of at least two processes that are both related to a key difference between the processing of speech produced by oneself and by somebody else, namely predictability. The speaker knows what word she is about to say and can thus anticipate hearing a particular word (arguably, one could consider this prediction a forward model). The listener, in contrast, cannot make such reliable predictions. In the specific context of our task, each word on the screen has a 0.25 probability of being spoken, which means that any prediction in perception is likely to be correct on only a minority of trials. In realistic situations, depending on context, the listener may sometimes have much better odds of predicting somebody's speech, but many other times the odds will be much worse. This difference between comprehension and production may have played out in our experiments in two ways.

We conjecture that one factor that contributes to a difference in visual attention in speech production and perception is a suppression of activation of the target in speech production. Evidence for such a suppression of activation comes for instance from MEG studies of word production (Heinks-Maldonado et al., 2006; Tian and Poeppel, 2011). During production upcoming words are predicted, followed by a suppression of activation of the predicted word. An interesting possibility is that such predictions result in early eye-movements to the phonological competitors (prediction) followed by a lack of activation-related effects (suppression). However, as noted above, there was no evidence for such early competition effects. But we do find a difference in magnitude of effects in production and perception. The suppression of activation of the target word could lead to decreased priming in production compared to perception, reflected by the decreased fixation proportions to the phonological competitor in production compared to perception.

In addition, lack of predictability in perception as compared to production may also affect phonological competition between cohort members. As in previous studies, results in the current experiment show that in perception trials the listener looks at the word with the closest correspondence to the word they hear. In the trial in which there is a phonological competitor word, which shares the onset, participants cannot be sure about which word will be the target until the uniqueness point has passed. The phonological competition is thus at least partly driven by uncertainty about the target word. In contrast, in the production trials the participant can predict which words she will hear herself say with almost complete certainty. Therefore any evidence for phonological competition in the eye gaze pattern is unlikely to reflect uncertainty about the target. Instead, we suggest that phonological competitor effects in production are due to activation spreading in the representational conglomerate that binds together the visuospatial and linguistic elements (Huettig et al., 2011): producing the phonology of one object's name at a particular spatial location primes the phonological representation of a related object as well as a pointer to its spatial location.

To conclude, the present results are most compatible with the view that eye-movements are driven similarly by the perception of one's own speech production and by the perception of someone else's speech. In both cases, overt, and not inner speech, drives the observed eye-movements. However, phonological competition effects in speech production and speech perception are also influenced by distinct processes as evidenced by much larger and much more long-lived phonological competitor effect in perception. In production trials, the target is strongly predicted and target activation is suppressed, whereas in perception, decreased target predictability results in phonological cohort competition. Thus, while it is correct that the channel we use to listen to ourselves and to someone else is the same (i.e., overt speech), because of a profound difference in predictability, the way we listen is different.

### Conflict of interest statement

The authors declare that the research was conducted in the absence of any commercial or financial relationships that could be construed as a potential conflict of interest.

## Footnotes

^1^Although it does not form part of this paper, we also successfully replicated the original Huettig and Hartsuiker (2010) experiment. A description of that experiment is available from the authors on request.

## Appendix A

**Table:**

**Semantic target**	**Competitor**	**Filler 1**	**Filler 2**
Mes *knife*	Kopje *cup*	Fiets *bicycle*	Vinger *finger*
Been *leg*	Pols *wrist*	Zeef *sieve*	Harp *harp*
Duim *thumb*	Oog *eye*	Schaal *bowl*	Bok *goat*
Teen *toe*	Arm *arm*	Water *water*	Lampion *lamp*
Nek *neck*	Lippen *lips*	Kam *comb*	Appel *apple*
Steen *stone*	Kei *boulder*	Wind *wind*	Computer *computer*
Bed *bed*	Stoel *chair*	Oorlog *war*	Lippenstift *lipstick*
Berg *mountain*	Rots *rock*	Netje *net*	Selder *celery*
Tent *tent*	Caravan *caravan*	Ananas *pineapple*	Saxofoon *saxophone*
Onweer *lightning*	Regen *rain*	Sokken *socks*	Broer *brother*
Trein *train*	Helikopter *helicopter*	Printer *printer*	Rog *ray*
Pan *pan*	Oven *oven*	Bank *sofa*	Vlag *flag*
Appelsien *orange*	Peer *pear*	Heuvel *hill*	Competitie *competition*
Perzik *peach*	Aardbei *strawberry*	Kano *canoe*	Broek *trousers*
Zon *sun*	Wolk *cloud*	Hoofd *head*	Radio *radio*
Hamer *hammer*	Boor *drill*	Jas *coat*	Mango *mango*
Glas *glass*	Kan *pitcher*	Hok *pen*	Batterij *battery*
Hark *rake*	Kruiwagen *wheelbarrow*	Droom *dream*	Lever *liver*
Nagel *nail*	Hand *hand*	Fles *bottle*	Salade *salad*
Ring *ring*	Horloge *watch*	Vis *fish*	Schilderij *painting*
Ketting *necklace*	Armband *bracelet*	Grond *soil*	Bot *bone*
Touw *rope*	Draad *thread*	Hemd *shirt*	Lam *lamb*
Tuinslang *hose*	Emmer *bucket*	Dag *day*	Servet *napkin*
Stropdas *tie*	Handschoen *glove*	Neef *nephew*	Plant *plant*
**Phonological target**	**Competitor**	**Filler 1**	**Filler 2**
Lamp *lamp*	Lam *lamb*	Kopje *cup*	Druif *pigeon*
Vinger *finger*	Vin *fin*	Caravan *caravan*	Poes *cat*
Hart *heart*	Harp *harp*	Appelsien o*range*	Nest *nest*
Bot *bone*	Bok *goat*	Perzik *peach*	Klok *clock*
Lat *latch*	Lampion *lamp*	Peer *pear*	Neus *nose*
Appel *apple*	Anker *anchor*	Hark *rake*	Bil *buttock*
Banaan *banana*	Batterij *battery*	Tuinslang *hose*	Raam *window*
Lepel *spoon*	Lever *liver*	Stoel *chair*	Gordijn *curtain*
Saxofoon s*axophone*	Salade *salad*	Helikopter *helicopter*	Kleed *rug*
Vlieger *kite*	Vlierstruik *elder*	Duim *thumb*	Spons *sponge*
Lippenstift *lipstick*	Lift *elevator*	Trein *train*	Hond *dog*
Rok *skirt*	Rog *ray*	Glas *glass*	Borstel *brush*
Riet *reed*	Riem *belt*	Tent *tent*	Kiwi *kiwi*
Broek *trousers*	Broer *brother*	Arm *arm*	Ketel *kettle*
Aardappel *potato*	Aap *monkey*	Rots *rock*	Haven *harbour*
Selder *celery*	Servet *napkin*	Teen *toe*	Vork *fork*
Gitaar *guitar*	Gieter *water can*	Horloge *watch*	Trap *stairs*
Vlag *flag*	Vlam *flame*	Hamer *hammer*	Telefoon *telephone*
Computer *computer*	Competitie *competition*	Kan *pitcher*	Maan *moon*
Radio *radio*	Radar *radar*	Ketting *necklace*	Mok *mug*
Schilderij *painting*	Schil *peel*	Touw *rope*	Kin *chin*
Man *man*	Mango *mango*	Handschoen *glove*	Weer *weather*
Plant *plant*	Plak *slice*	Wolk *cloud*	Zebra *zebra*
Bakker *baker*	Bakfiets *tricycle*	Aardbei *strawberry*	Zaag *saw*
**Neutral target**	**Filler 1**	**Filler 2**	**Filler 3**
Hond *dog*	Dag *day*	Steen *stone*	Lamp *lamp*
Poes *cat*	Droom *dream*	Boor *drill*	Vin *fin*
Vis *fish*	Grond *soil*	Onweer *thunderstorm*	Hart *heart*
Fles *bottle*	Maan *moon*	Kruiwagen *wheelbarrow*	Lat *latch*
Neus *nose*	Hemd *shirt*	Ring *ring*	Anker *anchor*
Kin *chin*	Hok *pen*	Zon *sun*	Banaan *banana*
Hoofd *head*	Jas *coat*	Bed *bed*	Lepel *spoon*
Bil *buttock*	Kano *canoe*	Regen *rain*	Vlieger *kite*
Raam *window*	Kiwi *kiwi*	Lippen *lips*	Bakker *baker*
Printer *printer*	Klok *clock*	Oven *oven*	Rok *skirt*
Gordijn *curtain*	Haven *harbour*	Pan *pan*	Vlierstruik *elder*
Kleed *rug*	Mok *mug*	Hand *hand*	Riet *reed*
Bank *sofa*	Neef *nephew*	Oog *eye*	Riem *belt*
Heuvel *hill*	Nest *nest*	Emmer *bucket*	Aardappel *potato*
Wind *wind*	Netje *net*	Mes *knife*	Aap *monkey*
Ananas *pineapple*	Oorlog *war*	Draad *thread*	Gitaar *guitar*
Druif *pigeon*	Sokken *socks*	Berg *mountain*	Gieter *water can*
Spons *sponge*	Telefoon *telephone*	Armband *bracelet*	Vlam *flame*
Borstel *brush*	Trap *stairs*	Nagel *nail*	Radar *radar*
Kam *comb*	Water *water*	Stropdas *tie*	Bakfiets *tricycle*
Ketel *kettle*	Zaag *saw*	Pols *wrist*	Man *man*
Schaal *bowl*	Weer *weather*	Nek *neck*	Plak *slice*
Zeef *sieve*	Vork *fork*	Been *leg*	Lift *elevator*
Fiets *bicycle*	Zebra *zebra*	Kei *boulder*	Schil *peel*

## Appendix B

**Table:** **Proportion of fixations in the phonological condition, measured in 200 ms timeframes, and statistical values of the comparison of fixation proportions to target and competitor against the unrelated items**.

**Start time of measurement relative to speech onset (ms)**		**Perception**					**Production**				
		**Mean**	***t***_**1**_	***p***	***t*2**	***p***	**Mean**	***t***_**1**_	***p***	***t*2**	***p***
−200	Target	0.246	−0.647	0.261	−0.609	0.275	0.957	93.100	0.000	81.338	0.000
	Competitor	0.245	−0.551	0.293	−0.509	0.308	0.014	−0.114	0.455	−0.122	0.452
	Unrelated	0.254					0.015				
0	Target	0.248	−0.129	0.449	−0.103	0.469	0.897	47.804	0.000	55.878	0.000
	Competitor	0.251	0.068	0.473	0.033	0.485	0.041	1.775	0.042	1.089	0.144
	Unrelated	0.250					0.031				
200	Target	0.268	1.388	0.087	1.076	0.147	0.815	23.035	0.000	36.292	0.000
	Competitor	0.259	1.182	0.122	1.071	0.148	0.076	1.717	0.047	2.445	0.012
	Unrelated	0.236					0.054				
400	Target	0.365	8.753	0.000	6.794	0.000	0.742	14.591	0.000	33.059	0.000
	Competitor	0.270	5.743	0.000	4.591	0.000	0.096	2.110	0.021	1.300	0.104
	Unrelated	0.182					0.080				
600	Target	0.524	22.674	0.000	19.134	0.000	0.700	12.533	0.000	32.661	0.000
	Competitor	0.247	7.904	0.000	5.832	0.000	0.100	0.018	0.493	0.056	0.478
	Unrelated	0.114					0.099				
800	Target	0.701	23.953	0.000	25.335	0.000	0.666	11.531	0.000	27.399	0.000
	Competitor	0.148	6.878	0.000	3.995	0.001	0.106	−0.490	0.314	−0.481	0.318
	Unrelated	0.075					0.113				
